# Processing of sensory, painful and vestibular stimuli in the thalamus

**DOI:** 10.1007/s00429-022-02582-y

**Published:** 2022-10-14

**Authors:** Kathrin Habig, Heidrun H. Krämer, Gothje Lautenschläger, Bertram Walter, Christoph Best

**Affiliations:** 1 Department of Neurology, Justus-Liebig-University, Klinikstrasse 33, 35392 Giessen, Germany; 2 Bender Institute of Neuroimaging, Justus-Liebig-University, 35394 Giessen, Germany; 3 Center for Mind, Brain and Behavior, Philipps University Marburg and Justus Liebig University, Giessen, Germany; 4 Department of Neurology, Philipps-University Marburg, 35043 Marburg, Germany

**Keywords:** Thalamus, Sensory perception, fMRI, Multimodal, Vestibular, Heat pain

## Abstract

**Objectives:**

The thalamus plays an important role in the mediation and integration of various stimuli (e.g., somatosensory, pain, and vestibular). Whether a stimulus-specific and topographic organization of the thalamic nuclei exists is still unknown. The aim of our study was to define a functional, in vivo map of multimodal sensory processing within the human thalamus.

**Methods:**

Twenty healthy individuals (10 women, 21–34 years old) participated. Defined sensory stimuli were applied to both hands (innocuous touch, mechanical pain, and heat pain) and the vestibular organ (galvanic stimulation) during 3 T functional MRI.

**Results:**

Bilateral thalamic activations could be detected for touch, mechanical pain, and vestibular stimulation within the left medio-dorsal and right anterior thalamus. Heat pain did not lead to thalamic activation at all. Stimuli applied to the left body side resulted in stronger activation patterns. Comparing an early with a late stimulation interval, the mentioned activation patterns were far more pronounced within the early stimulation interval.

**Conclusions:**

The right anterior and ventral-anterior nucleus and the left medio-dorsal nucleus appear to be important for the processing of multimodal sensory information. In addition, galvanic stimulation is processed more laterally compared to mechanical pain. The observed changes in activity within the thalamic nuclei depending on the stimulation interval suggest that the stimuli are processed in a thalamic network rather than a distinct nucleus. In particular, the vestibular network within the thalamus recruits bilateral nuclei, rendering the thalamus an important integrative structure for vestibular function.

## Introduction

In recent years, the concept of the salience detection network has been established (Legrain et al. 2011). Since then, imaging techniques could reassign networks for the processing of pain, memory, tactile stimulation, and so forth. Within the salience detection network, the thalamus in collaboration with the insular cortex and the anterior cingulate cortex play crucial roles in the integration of salient information (Zhou et al. 2021).

There are numerous studies about the features and function of specific thalamic nuclei derived by lesion models, ante- and retrograde tracing techniques as well as electrophysiological analysis (Mo and Sherman 2019; Raymond et al. 1974; Xue et al. 2022).

In humans, evidence is much more limited. Analysis of the functional specialities relies upon imaging studies in healthy subjects, case analysis of patients with epilepsy or lesion studies in patients with structural damage of the thalamic region (for review see Blomqvist et al. 2000; Guido and Huberman 2022).

Behrens and coworkers performed the first connectivity-based segmentation of the human thalamus and showed seven latero-caudal-oriented areas (Behrens et al. 2003). Johansen-Berg et al. (2005) and later Kumar et al. (2015) provided a parcellation of the thalamus by diffusion-weighed imaging techniques.

The approved doctrine about thalamic organization assumes an anatomical and congruent functional division in thalamic nuclei. The ventral posteriolateral nucleus [VPL] and ventral posteriomedial nucleus [VPM]) are known as somatosensoric nuclei (Krause et al. 2012). And especially the ventral medial thalamic nucleus (VMpo) is supposed to mediate heat and pain (Craig et al. 1994). Furthermore, thalamic activation has been observed during the experience and cognitive modulation of pain (Tracey 2010), muscle pain (Zimmermann et al. 2012), heat pain [see meta-analysis by Lanz et al. (2011)], as well as during the imagination of pain (Krämer et al. 2008).

The processing of visual data within the thalamic lateral geniculate nuclei has been investigated extensively (Díaz et al. 2018; Kremkow and Alonso 2021; Kurzawski et al. 2020).

Furthermore, it has been shown, that the thalamus is an important part of the vestibular system. In functional MRI studies, caloric as well as galvanic vestibular stimulation evoked activation of thalamic nuclei, in particular, the posteriolateral and posteriomedial nuclei (Bottini et al. 2001; Dieterich et al. 2003; Marcelli et al. 2009). Therefore, an overlap of vestibular and nociceptive nuclei was discussed. Lesion studies have confirmed the importance of the posteriolateral nuclei in the processing of vestibular stimuli (Dieterich and Brandt 1993). In a recent review, the authors propose that the bilateral projections from the brainstem to the thalamus are the basis for lateralization of certain brain functions (e.g., vestibular stimulation or hand performance and spatial orientation (Brandt and Dieterich 2019).

In the last decade, another concept of thalamic function has been introduced: Kumar et al. (2015) proposed that the thalamus has a “central core” function in guiding attention and processing of sensory stimuli because the defined clusters all showed similar cerebral connections. Thalamic networks have been found for executive, language and memory functions (Hwang et al. 2021) and by genetic gradients in the thalamus as well as histological and molecular studies questioning the traditional nuclei boundaries (Halassa and Murray Sherman 2019; Xue et al. 2022).

Recent imaging studies did not focus on intrathalamic connections and some even discarded those from their analysis (e.g. Grodd et al. 2020).

Until now it has not been investigated whether a multimodal integration of distinct afferent signals exists within the thalamus. We intended to study various sensory stimuli applied to a preferably small anatomical area (filaments/pin prick devices with a very small calibre on the fingertip for touch and pain and thermal stimulation at the same hand). Additionally, we aimed to investigate another sensory input, which does not derive from the skin and can be achieved reliably. Therefore, we decided on vestibular stimulation. From the different possibilities of vestibular stimulation, e.g., caloric or galvanic vestibular stimulation, we decided on galvanic vestibular stimulation. Applying galvanic vestibular stimulation yields various advantages: Galvanic vestibular stimulation results in whole nerve stimulation, including the semicircular canal as well as the otolith afferents, while a caloric vestibular stimulation activates the semicircular canal afferents only. Furthermore, galvanic vestibular stimulation -besides the vestibular stimulation- does evoke pain stimulation at the site of the electrodes. This area can be anaesthetised so that the resulting activation is purely vestibular. A caloric vestibular stimulation, however, will always consist of additional stimulatory components, as the effect from the thermal convection cannot be antagonized. Finally, the galvanic stimulation displays the more safe stimulatory way inside the MRI scanner.

Summarizing, we investigated the processing of (a) sensory stimuli (touch), (b) nociceptive stimuli (heat and mechanical pain), and (c) vestibular stimuli. This choice of stimuli is comparable to the ones used in a functional map of the operculo-insular cortex (zu Eulenburg et al. 2013).

The aim of the study was to define the thalamic activation patterns by multimodal stimuli and therefore contribute to an in vivo mapping of the human thalamus.

## Methods

### Participants

Twenty healthy individuals participated in the study. After preprocessing all MR images, three participants were excluded from further analysis because their images did not meet the quality criteria. Nine women (mean age 28.4 ± 1.6) and eight men (27.1 ± 1.6 years old) remained in the assessment and were included in the following analyses and results. The medical history and the clinical neurological examination were unremarkable in all participants. All participants were fully right-handed according to the modified handedness score (Varney and Benton 1975).

All participants gave their informed written consent according to the latest revision of the Declaration of Helsinki. The study was approved by the local ethics committee of the Justus Liebig University Giessen (37/14).

### Stimulation

#### Heat pain

Heat pain thresholds (HPTs) were determined in all participants using a TSA 2001-II thermode (MEDOC, Israel) at the palm of the hand (baseline temperature: 32 °C; contact area of the thermode: 9.0 cm^2^, ramp rate: 1 °C/s). The mean HPT was calculated from three consecutive measurements according to the method of limits (for detail see, Rolke et al. 2006). The individual HPT was acquired directly before the subject entered the scanner. To overcome temperature changes and habituation of heat pain, the temperature applied in the scanner was 2 °C above the individual threshold. The mean HPT was 44.4 °C (± 1.1 °C SEM). The mean temperature used during the fMRI experiment was 46.8 °C (± 0.3 °C SEM).

#### Tactile stimulation

Tactile detection was tested before the fMRI experiment with pinprick devices (the PIN PRICK, SenseLab; non-magnetic devices ranging from 8 to 512 mN). To obtain a reliable mechanical stimulus, touch with a pinprick device was applied on the finger pad of the middle finger for 14 s. All participants rated a tactile stimulus, with 8 mN and 16 mN as noticeable and painless. Tactile stimulation was therefore performed using the 16-mN pinprick device.

#### Mechanical pain

Pinprick devices were also used to examine the mechanical pain threshold (MPT). The mean MPT was calculated by applying ascending and descending pinprick forces according to the method of limits (for detail see Rolke et al. 2006). For the fMRI experiment, the next higher pinprick device was used (available forces: 8, 16, 32, 64, 128, 256, and 512 mN). The pinprick device was applied to the same location as the tactile stimulus was (finger pad of the middle finger) with constant contact throughout the trial. MPT was found to be 240.9 mN (mean; SEM 30.32 mN). To avoid adaptation, the next stronger pinprick device was used during fMRI (mean: 406.6 mN; SEM 36.79 mN).

#### Galvanic stimulation

For galvanic stimulation, two carbon electrodes were adhered to the mastoids bilaterally. To minimalize sensory input, the skin of both mastoids was anesthetized with lidocaine cream. The stimulation is capable of evoking electric impulses ranging from − 4 to + 4 mA to each mastoid. The frequency of that periodic applied current (alternating and direct current) is low (1 Hz) with a duration of 0.5 s. The stimulation has been carried out unilaterally. This technique is used to detect dysfunction of the vestibular nerve or vestibular hair cells in specialized laboratories (Jahn et al. 2003). By applying current to each mastoid in an alternating manner, the vestibular input is not simultaneous and it evokes the illusion of vertigo. It relies on the neurophysiological investigations of the electric characteristics of the vestibular system by Goldberg et al. (1984). Before the fMRI session, the electrical threshold to elicit constant vertigo was investigated in each subject. For the fMRI experiment, the stimulation was carried out with the individual’s threshold + 1 mA. When pain has been reported in a standardized questioning after the stimulation the session has been discarded from the analysis. Galvanic stimulation evoked light dizziness with 1.35 mA (mean, SEM 0.15 mA). Galvanic stimulation was performed with 1.9 mA (mean; SEM 0.23 mA) during the experimental session.

### Study design

We employed a within-subject design. The stimuli were applied constantly in blocks of 14 s duration. Each block was immediately followed by the next type of stimulation in a constant order: heat, touch, galvanic stimulation, pain. The fixed order was chosen to keep the frequency of stimulation regressors constant. This ensures that the high pass filter affects all regressors in the same fashion. Additional breaks between stimulations would reduce the regressor’s variances and—therefore—degrade the efficiency of contrasts of interest. This sequence (heat, touch, galvanic stimulation, pain) was repeated ten times with a resting phase of 14 s between repetitions. As described in “Data preprocessing” we discarded the two first images (2.8 s) and added 17 s at the end of the registration for the last bold sequence to be finished, resulting in a session duration of 720 s. Stimuli were applied to the left and the right sides of the subject in successive sessions in random order.

This design was tested for efficiency using the FMRI Expert Analysis Tool (FEAT) Version 6.00, part of FSL (FMRIB’s software library, www.fmrib.ox.ac.uk/fsl), which reported that for the simple contrasts of the stimulation signal, a change of only 1.2% is sufficient to obtain *z* > 5.3.

### fMRI

#### Data acquisition

All MR images were acquired with a Siemens Prisma 3 T scanner (Siemens, Erlangen, Germany). For anatomical imaging, an MPRAGE sequence (TE = 2.3 ms, PAT = 3, matrix size = 176 × 256 × 256, voxel size 0.9 x 0.9 x 0.9 mm^3^, field of view 165 × 240 × 240 mm) was used. Functional images were acquired with a Siemens Zoomit EPI sequence (400 volumes, TR = 1.8 s, TE = 31 ms, flip angle = 90°, matrix size = 94 × 30, 24 adjacent 2-mm thick slices with 0.5 mm gap in descending acquisition, voxel size = 2 × 2 × 2 mm^3^, field of view 188 × 60 × 60 mm, centred on the thalamus). Additionally, a whole-brain functional scan was obtained with an EPI sequence (5 volumes, TR = 3.5 s, TE = 30 ms, flip angle = 90°, matrix size = 94 × 94, 58 adjacent 2-mm thick slices with 0.5 mm gap in descending acquisition, voxel size = 2 × 2 × 2 mm^3^, field of view 188 × 188 × 145 mm) for registration purposes. To account for B0 inhomogeneity, a field map was measured with a double echo sequence (TE1 = 10 ms, TE2 = 12.46 ms, flip angle = 90°, voxel size = 2 × 2 × 2.5 mm^3^, field of view 220 × 220 × 150 mm).

#### Data preprocessing

The first three volumes of the measurements were automatically not recorded by the scanner. We decided to remove an additional two volumes, as we could show in previous test scans that the saturation of the signal is clearly in a steady state from the third scan on. After that, the following steps were performed: motion correction (with FSL’s MCFLIRT by maximizing the normalized correlation between each time point and final spline interpolation), unwarping of B0 distortions (using field map images with FSL’s FEAT), registration to MNI standard space (six-parameter rigid body registration of thalamus volumes to whole-brain volumes with FSL’s FLIRT, six-parameter rigid body registration of whole-brain volumes to anatomical images with FSL’s FLIRT using boundary-based registration, 12-parameter affine registration of anatomical volumes to FSL’s standard MNI152_T1_2mm_brain further refined using FSL’s FNIRT nonlinear registration), smoothing using FSL’s SUSAN with a kernel of 5 mm, and high-pass filtering with a cut-off of 100 s.

To identify deformed functional volumes due to motion during a volume scan, each volume was compared with its two neighbours in the motion-corrected time series by calculating the mean square differences. The smaller difference was used as the measure for the outlier value for each volume. The scores were set within a threshold according to the method of Hubert and van der Veeken (2008), and volumes above the threshold were treated as outliers in subsequent analyses.

Finger pulse data were processed with the Physio-Toolbox (Version r671; http://www.translationalneuromodeling.org/tnu-checkphysretroicor-toolbox/) to obtain six RETROICOR regressors (Glover et al. 2000) and another regressor based on the cardiac response function (Chang et al. 2009).

Voxels containing only cerebrospinal fluid were identified by segmentation of anatomical images using FSL’s FAST. Partial volume estimates for CSF masked with a brain mask were resampled to functional volumes and set within a threshold that contains only voxels in the ventricles with a CSF probability of 1. The first five eigenvalues of the time series of these voxels extracted with FSLMEANTS served as CSF regressors.

#### Statistical modelling and inference

Each session of the 17 remaining subjects was analysed with a multiple regression model using FSL’s FILM with local autocorrelation correction (Woolrich et al. 2001). The design consisted of the following regressors: type of stimulation (touch, heat, galvanic stimulation, and pain) convolved with FSL’s double-gamma HRF and the first derivatives of these regressors as well as up to two additional regressors for miscarried stimulation events (convolved with HRF and their first derivatives, six motion regressors, seven regressors for cardiac-based signal, five CSF regressors, and an additional regressor for each outlying volume). All regressors were high-pass filtered with a cut-off of 100 s. Contrasts were calculated for the main regressors of stimulus types only. Additionally, we defined two times (early = 0–3 s of the stimulation block; late = 3–14 s of the stimulation block), orthogonalized the later regressors with regard to the first ones, and carried out session-level analyses with these now-split regressors. The time intervals have been chosen to overcome habituation processes of heat pain (Treede et al. 1995) and to illustrate time-dependent activations according to zu Eulenburg et al. (2013) and Pomares et al. (2013).

Group analyses used a design for repeated measures ANOVA with the factors’ stimulation type and side of stimulation. Voxel-intensity-based inferences were computed by permutation tests using PALM (version alpha115; https://fsl.fmrib.ox.ac.uk/fsl/fslwiki/PALM; (Winkler et al. 2014) on GNU Octave (version 4.0.3) with 500 permutations and tail approximation using a generalized Pareto distribution for *p* values (Winkler et al. 2016) and a familywise error corrected for the number of voxels tested. All analyses were performed for thalamus voxels only, using a thalamus mask constructed by the addition of the left and right thalamus masks from the Harvard Oxford subcortical atlas delivered with FSL. This mask contains all voxels that belong to the thalamus with a higher probability than to any other region.

First, activation of each stimulation at each side was assessed for the whole duration of stimulation as well as for early and late stimulation separately using voxel-level tests to ensure the exact location of significant voxels. Early and late stimulation periods were compared by the more powerful threshold-free cluster enhancement (TFCE, (Smith and Nichols 2009). Because two tests were performed for each stimulation, a Bonferroni correction was applied resulting in *α* = 0.025.

Anatomic labelling of the local maxima of activations within the thalamus was performed with the aid of the Thalamic Nuclei Atlas (Najdenovska et al. 2018) and the Oxford thalamic connectivity atlas (Behrens et al. 2003) delivered with FSL.

The Thalamic Nuclei Atlas is based on the parcellation by Battistella et al. (2017). Battistella et al. incorporated the anatomic labelling of Morel et al. (1997). Table 1 is supposed to assign the anatomic labels by Morel to the parcellation of the Thalamic Nuclei Atlas.

**Table 1:** Anatomic labelling

Battistella 2017	Morel 1997
A	AM, AV, AL
VA	VA
MD	CeM, MD_pc_, MD_pl_, CL,
VLV	VPM, VPL_p_, VPL_a_, VLp_v_, VLa
VLD	VLp_d_, LP (lateral part)
CL-LP-PuM	CL, LP (medial part), PuM (medial part)
Pu	PuM, PuA, PuL

*A* anterior, *VA* ventral-anterior, *MD* medio-dorsal, *VLV* ventral latero-ventral, *VLD* ventro-larteero-dorsal, *CL-LP-PuM* central-lateral/lateral-posterior/medial-pulvinar, *Pu* pulvinar, *AM* antero-medial, *AV* antero-ventral, *AL* antero-lateral, *CeM* central medial, *MD*_*pc*_ mediodorsal nucleus, parvocellular division, *MD*_*pl*_ mediodorsal nucleus, paralamellar division, CL central lateral, VPM ventral posterior medial, VPL_p_ ventral posterior lateral nucleus, posterior division, *VPL*_*a*_ ventral posterior lateral nucleus, anterior division, *VLp*_*v*_ ventral lateral posterior nucleus, ventral division, *VLa* ventral lateral anterior, *VLp*_*d*_ ventral lateral posterior nucleus dorsal division, *LP* lateral posterior, *PuM* medial pulvinar, *PuA* anterior pulvinar, *PuL* lateral pulvinar

## Results

### Activation by different stimuli during early phases of stimulation

**Fig. 1:**
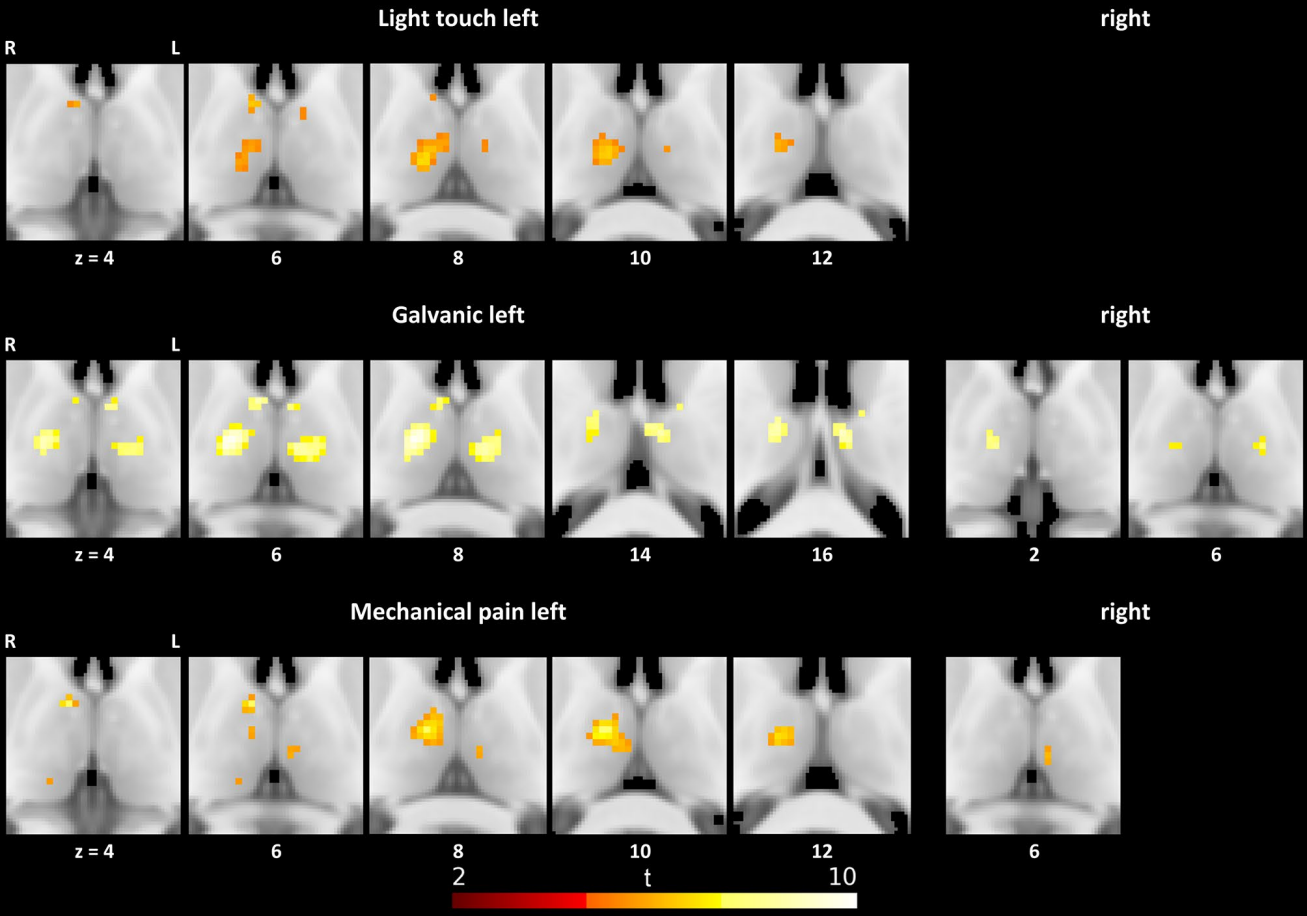
Thalamic responses to early (1–3 s) stimulation

Considering two-time intervals with an early phase (T1 = 0–3 s) and a late phase (T2 = 4–14 s), we found considerably more BOLD responses in the early phase for light touch, galvanic stimulation, and mechanical pain (see Table 2 and Fig. 1).

**Table 2:** Significant local maxima of thalamic responses to early (1–3 s) and late (4–14 s) stimulation: size of clusters of significant voxels, MNI coordinates and voxel level tests

Stimulation, time frame Side	Cluster size (*n* voxels)	*x*	*y*	*z*	*t*	*p*_FWE_^a^	TNA	OTCA
Light touch, early								
Left	60	12	− 20	8	6.742	< 0.001	Right VLD	PFC
	7	8	− 2	6	6.409	0.001	right A	PFC
	3	− 8	− 16	10	5.536	0.013	Left MD	PFC
	2	− 8	− 6	6	5.341	0.021	Left A	PFC
Right						n.s		
Light touch, late								
Left						n.s		
Right						n.s		
Galvanic, early								
Left	92	14	− 14	8	9.890	0.001	Right VA	PFC
		14	− 10	16	8.704	0.004	Right VA	PFC
	56	− 8	− 18	8	8.615	0.004	Left MD	PFC
	19	− 6	− 12	16	9.024	0.003	Left VA	TC
		− 2	− 10	14	8.191	0.007	–	TC
	10	6	− 2	6	8.716	0.004	Right A	TC
	5	− 4	− 4	4	8.173	0.007	Left A	TC
	2	− 12	− 4	14	7.442	0.016	Left VA	PFC
Right	14	12	− 16	2	8.173	< 0.001	Right VLV	PFC
	5	− 14	− 16	6	7.576	0.005	Left MD	PreMC
Galvanic, late								
Left	1	18	− 20	8	2.815	0.015	Right VLV	PriMC
Right						n.s		
Heat pain, early								
Left						n.s		
Right						n.s		
Heat pain, late								
Left						n.s		
Right						n.s		
Mechanical pain, early								
Left	73	12	− 12	10	7.713	0.001	Right VA	PFC
		8	− 4	6	7.356	0.002	Right A	PFC
	5	− 4	− 20	6	5.965	0.014	Left MD	PFC
	2	14	− 28	4	5.652	0.023	Right P	PPC
Right	3	− 4	− 20	6	6.164	0.008	Left MD	PFC
Mechanical pain, late								
Left	5	2	− 20	12	2.632	0.007	–	TC
	4	− 2	− 20	6	2.584	0.010	Left MD	PFC
Right						n.s.		

*TNA* thalamic nuclei atlas, *A* anterior, *CL* central-lateral/lateral-posterior/medial-pulvinar, *MD* medio-dorsal, *P* pulvinar, *VA* ventral-anterior, *VLD* ventral-latero-dorsal, *VLV* ventral-latero-ventral, *OTCA* oxford thalamic connectivity atlas, *OC* occipital cortex, *PFC* pre-frontal cortex, *PPC* posterior parietal cortex, *PreMC* pre-motor cortex, *PriMC* primary motor cortex, *SC* sensory cortex, *TC* temporal cortex

^a^As two tests are performed for each stimulus type and time frame (two stimulation sides) the significance threshold according to Bonferroni is set to *α* = 0.025

Light touch to the left hand elicited early activation in the right ventral-latero-dorsal nucleus as well as the anterior nuclei bilaterally and the left medio-dorsal nucleus.

Galvanic stimulation (sinistral application) in the early phase showed activated clusters covering the ventral-anterior nucleus, medio-dorsal nucleus, and anterior nucleus.

Early activations by mechanical pain (sinistral application) were found in the left medio-dorsal nucleus.

Heat pain stimulation did not elicit activated voxels in the early phase of acquisition.

The activations by 14 s of stimulation are shown in Table 4.

### Activation by different stimuli during late phases of stimulation

**Fig. 2:**
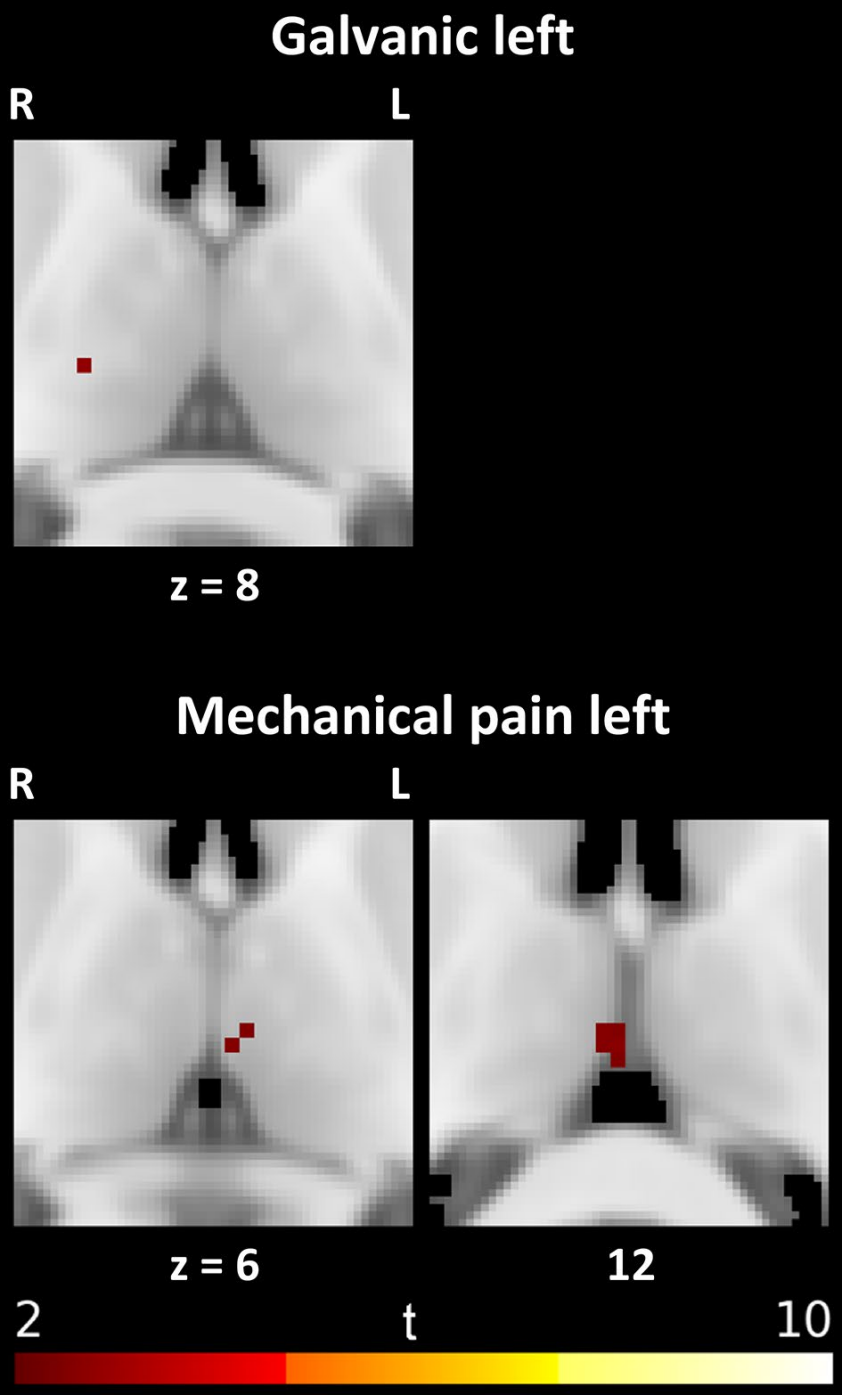
Thalamic responses to late (4–14 s) stimulation

During galvanic stimulation, the late-phase activation could be assigned to only one voxel in the right ventral-latero-ventral nucleus (see Fig. 2).

Mechanical pain to the left showed activations of the left medio-dorsal nucleus and unassigned activations near the interhemispheric space.

Light touch and heat pain elicited no activation in the late phase.

Comparing both time intervals, early activations exceeded late responses to light touch, galvanic stimulation, and mechanical pain (see Table 3). No stimulation yielded higher responses in the late phase.

**Table 3:** Local maxima of early (1–3 s) vs. late (4–14 s) thalamic responses to stimulation: cluster sizes, MNI coordinates and cluster level tests using threshold-free cluster enhancement (TFCE)

Stimulation, change Side	Cluster size (*n* voxels)	*x*	*y*	*z*	TFCE	*p*_FWE_^a^	TNA	OTCA
Light touch, early > late								
Left	239	10	− 20	8	361.942	0.001	Right CL	PFC
		8	− 2	6	264.139	0.003	Right A	PFC
	82	− 8	− 6	6	209.347	0.007	Left A	PFC
		− 8	− 16	10	186.919	0.011	Left MD	PFC
	8	16	− 20	2	156.542	0.021	Right VLV	PreMC
Right						n.s.		
Light touch, late > early								
Left						n.s.		
Right						n.s.		
Galvanic, early > late								
Left	830	14	− 14	8	856.675	0.001	Right VA	PFC
		6	− 2	6	719.046	0.001	Right A	TC
		14	− 10	16	715.389	0.001	Right VA	PFC
		6	− 8	10	686.752	0.001	Right A	TC
		− 6	− 12	14	658.412	0.002	Left VA	TC
		− 6	− 18	8	624.655	0.002	Left MD	PFC
Right	106	14	− 16	0	392.678	0.010	Right VLV	PreMC
		12	− 8	8	336.370	0.018	Right VA	PFC
		14	− 14	12	336.090	0.018	Right VA	PFC
		14	− 6	14	335.359	0.018	Right VA	PFC
		12	− 22	12	325.537	0.020	Right VLD	PFC
		8	− 2	4	317.749	0.022	Right A	PFC
	4	− 12	− 16	4	311.298	0.024	Left MD	PFC
	3	− 10	− 16	14	312.987	0.024	Left VA	PFC
Galvanic, late > early								
Left						n.s		
Right						n.s		
Heat pain, early > late								
Left						n.s		
Right						n.s		
Heat pain, late > early								
Left						n.s		
Right						n.s		
Mechanical pain, early > late								
Left	187	12	− 12	10	289.442	0.001	Right VA	PFC
		8	− 4	4	270.442	0.001	Right A	PFC
		6	− 6	− 6	142.883	0.016	–	–
Right						n.s		
Mechanical pain, late > early								
Left						n.s		
Right						n.s		

*TNA* thalamic nuclei atlas**,** *A* anterior, *CL* central-lateral/lateral-posterior/medial-pulvinar, *MD* medio-dorsal, *P* pulvinar, *VA* ventral-anterior, *VLD* ventral-latero-dorsal, *VLV* ventral-latero-ventral, *OTCA* oxford thalamic connectivity atlas, *OC* occipital cortex, *PFC* pre-frontal cortex, *PPC* posterior parietal cortex, *PreMC* pre-motor cortex, *PriMC* primary motor cortex, *SC* sensory cortex, *TC* temporal cortex

^a^As two tests are performed for each stimulus type and time frame (two stimulation sides) the significance threshold according to Bonferroni is set to *α* = 0.025

### Activation by different stimuli during the complete stimulation phase

**Fig. 3:**
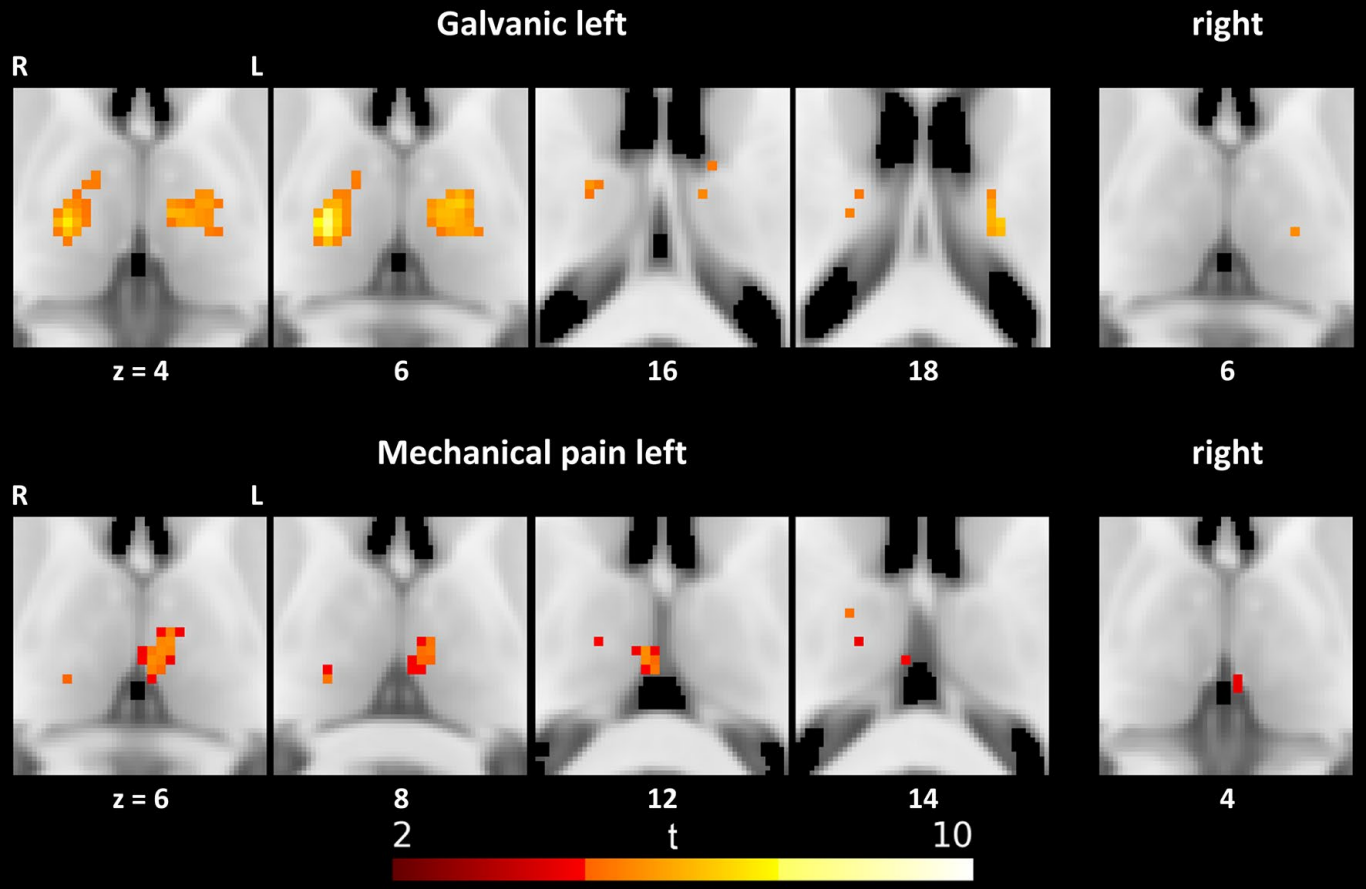
Thalamic responses to 14 s stimulation

Remarkable bilateral activation during the complete stimulation phase of 14 s was found by galvanic stimulation and mechanical pain (see Table 4 and Fig. 3). No significant activation could be shown during touch and heat stimulation in the thalamus.

**Table 4:** Significant local maxima of thalamic responses to 14 s stimulation: size of clusters of significant voxels, MNI coordinates and voxel level tests

Stimulation side	Cluster size (*n* voxels)	*x*	*y*	*z*	*t*	*p*_FWE_^a^	TNA	OTCA
Light touch								
Left						n.s		
Right						n.s		
Galvanic								
Left	70	− 12	− 14	6	6.512	0.002	Left VA	PFC
	64	16	− 18	6	7.973	< 0.001	Right VLV	PreMC
		10	− 8	4	5.193	0.016	Right A	PFC
	8	− 16	− 18	18	6.469	0.002	Left VLD	PFC
	5	16	− 10	16	5.542	0.009	Right VA	PFC
	1	− 8	− 12	16	5.313	0.013	Left VA	TC
	1	− 10	− 6	16	5.056	0.021	Left VA	PFC
	1	16	− 16	18	5.209	0.016	Right VLD	PFC
Right	1	− 14	− 20	6	5.397	0.023	Left VLV	PreMC
Heat pain								
Left						n.s		
Right						n.s		
Mechanical pain								
Left	32	− 6	− 16	4	5.481	0.001	Left MD	PFC
	15	4	− 18	12	5.368	0.001	Right MD	TC
	3	16	− 24	8	4.971	0.005	Right VLD	PPC
	2	14	− 16	12	4.625	0.015	Right VA	PFC
	2	16	− 10	14	4.921	0.006	Right VA	PFC
Right	2	− 2	− 24	4	4.302	0.016	–	PFC

*TNA* thalamic nuclei atlas**,** *A* anterior, *CL* central-lateral/lateral-posterior/medial-pulvinar, *MD* medio-dorsal, *P* pulvinar, *VA* ventral-anterior, *VLD* ventral-latero-dorsal, *VLV* ventral-latero-ventral, *OTCA* Oxford thalamic connectivity atlas, *OC* occipital cortex, *PFC* pre-frontal cortex, *PPC* posterior parietal cortex, *PreMC* pre-motor cortex, *PriMC* primary motor cortex, *SC* sensory cortex, *TC* temporal cortex

^a^As two tests are performed for each stimulus type and time frame (two stimulation sides) the significance threshold according to Bonferroni is set to *α* = 0.025

Galvanic stimulation to the left side showed activated clusters mainly in the left ventral-anterior nucleus as well as a right ventral-latero-ventral nucleus and right anterior nucleus. Smaller clusters were activated in the ventral-latero-dorsal nucleus bilaterally. Right-sided stimuli elicited activations in the left ventral-latero-ventral nucleus.

Mechanical pain applied to the left hand showed the largest clusters in the medio-dorsal nuclei bilaterally, right ventral-latero-dorsal, and right ventral-anterior nuclei. Right-sided stimulation showed BOLD responses in only one single cluster near the interhemispheric space.

Thus, thalamic areas that have been activated by galvanic stimuli are located more laterally and ventrally than areas that can be assigned to activation by mechanical pain are.

Overlap of BOLD responses to different stimulations.

**Fig. 4:**
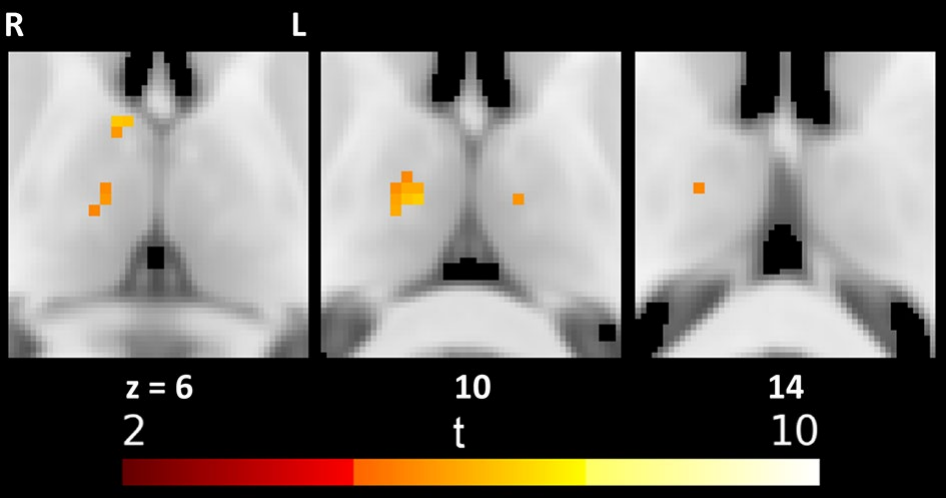
Conjunction of thalamic responses to early (1–3 s) left side stimulation with light touch, galvanic stimulation and mechanical pain

**Fig. 5:**
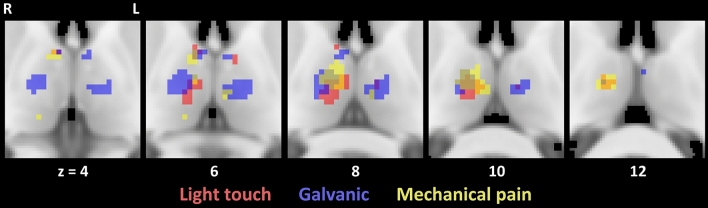
Overlap of thalamic responses to early (1–3 s) stimulation on the left side

All stimuli applied to the left body side (except heat pain) showed overlapping BOLD responses in the right anterior and ventral-anterior nucleus as well as in the left medio-dorsal nucleus (see Tables 2, 5 and Figs. 4 and 5).

**Table 5:** Significant local maxima of conjunction of thalamic responses to early (1–3 s) left side stimulation with light touch, galvanic stimulation and mechanical pain: size of clusters of significant voxels, MNI coordinates and voxel level tests

Cluster size (*n* voxels)	*x*	*y*	*z*	*t*	*p*_FWE_^a^	TNA	OTCA
20	12	− 14	10	5.926	0.005	Right VA	PFC
	16	− 14	14	5.314	0.024	Right VA	PFC
4	6	− 2	6	6.322	0.004	Right A	TC
3	− 8	− 16	10	5.536	0.014	Left MD	PFC

^a^As two tests are performed for each stimulus type and time frame (two stimulation sides) the significance threshold according to Bonferroni is set to *α* = 0.025

## Discussion

We found that thalamic activation appears to be less dependent on the type of stimulation but differs in aspects of stimulus duration. Heat pain stimulation did not result in thalamic activations. All other stimuli elicited bilateral activations in the human thalamus that mainly overlapped, referring to a possible multimodality of thalamic nuclei. Nevertheless, we also observed a slight topographic order with thalamic activation by galvanic stimulation lateral and ventral to mechanical pain. We observed a left hemispheric dominance of BOLD responses when the whole stimulation (14 s) was measured and a right hemispheric dominance during the first seconds of stimulation. Overall, we detected a preponderance of activations during the first seconds of the stimuli. Galvanic stimulation elicited the largest and most distributed activated clusters within the thalamus. Our results point to the importance of the thalamus in integrating early vestibular and sensory information.

### Innocuous touch

Innocuous touch is mainly processed in the contralateral ventro-lateral thalamus during the early time interval (first 1–3 s of the stimulation). Protopathic information derived from the fasciculus cuneatus are expected to enter the thalamus more ventrally, in the contralateral VPL. The distal body parts are represented more ventrally in the VPL (Loutit et al. 2020). Activation of the VPL could be established in the comparison of early and late stimulations. Early left-sided stimulation exceeded late stimulation primarily in the right CL (covering the central lateral nucleus as well as the lateral posterior and medial part of the pulvinar), bilateral anterior nuclei, and right ventral-latero-ventral nuclei (covering the VPL). Because of the overall small thalamic activation, it can be argued that the thalamus redistributes the information of innocuous touch to other brain areas where further processing occurs (Behrens et al. 2003; Romo and Rossi-Pool 2020; Vázquez et al. 2012).

### Thalamic integration of vestibular function

Galvanic stimulation showed the largest activated cluster in the left ventral-anterior nuclei and a much smaller cluster in the right ventral-latero-ventral nucleus (VLV). The thalamic activations occurred during the entire stimulation time, but they were pronounced in the early compared to the late phase. The mentioned nuclei are described as targets of major vestibulo-thalamic projections: the medial vestibular nucleus projects to the ventral posterior nucleus (including VPL, VPM, and VI; these nuclei are summarized as VLV following the nomenclature of the thalamic nuclei atlas; for comparison see Table 1) and the superior vestibular nucleus is supposed to project to the ventral posterior and ventral anterior nuclei of the thalamus (Brandt and Dieterich 2019; Kirsch et al. 2016; Wijesinghe et al. 2015). From the thalamic nuclei, the main path of vestibular information derived from animal studies and confirmed in humans leads to the cortical parieto-insular vestibular cortex (PIVC), known to be a core of integrating vestibular information and equipped with multisensory neurons, including the human operculo-insular cortex (Akbarian et al. 1992; Dieterich et al. 2003; zu Eulenburg et al. 2013). Our findings of bilateral BOLD responses during galvanic stimulation are in accordance with previous studies showing bilateral activation of the thalamus (Bense et al. 2001; Bucher et al. 1998) and of the operculo-insular cortex by vestibular stimulation (zu Eulenburg et al. 2013).

For human beings, a dominance of the right thalamic input of vestibular stimulations has been proposed. Dieterich and colleagues proposed a preponderance of right-sided galvanic stimuli in right-handed subjects in the right thalamus for the PIVC (Dieterich et al. 2003), and more recently, they confirmed a dominant right thalamic input of vestibular information in an investigation with DTI (Dieterich et al. 2017). In rat models, a left hemispheric dominance of vestibular input, independent of the handedness, has been described (Best et al. 2014).

Regarding the entire time interval of stimulation, we cannot support this observation. However, in the early phase, right-sided stimulation evoked more ipsilateral voxels (right thalamus), and during left-sided stimulation, contralateral voxel (right thalamus) prevailed. Therefore, for the early phase, we can reproduce the thesis that the right thalamus is dominant in right-handed subjects. We found an activation of nuclei that can be attributed to the known vestibulo-thalamic pathways bilaterally.

### Mechanical pain

Mechanical pain with a pinprick device well above the individual pain threshold evoked bithalamic activations when stimulating the left hand but only contralateral activations during right-sided stimulation. The activations were pronounced in the bilateral medio-dorsal nuclei, right ventral-latero-dorsal, and ventral-anterior nuclei.

The medio-dorsal nucleus and the ventral-latero-dorsal nucleus have distinct connections (derived from diffusion tractography) to the prefrontal cortex (Johansen-Berg et al. 2005), which is frequently activated during nociceptive stimuli (mechanical pain; non-experimental pain and less during heat pain (Lanz et al. 2011). Pain fibres from the spinothalamic tract are supposed to reach the ventrolateral thalamus via the brainstem and from there to the primary and secondary somatosensory cortex as well as the primary motor cortex (Craig 2002).

Previous histologically and electrophysiologically based studies investigating mechanical pain have shown that most nociceptive information reaches the VMpo via the spinothalamic tract (Blomqvist et al. 2000; Craig et al. 1994). Transferring the localization of VMpo to the nomenclature in this publication, the VMpo can be best attributed to the medio-dorsal and CL nucleus (it is situated ventrally to the anterior pulvinar and contacts anteriorly the VPM and medially the VM [posterior part]), referring to Blomqvist et al. (2000). The mediodorsal nucleus concordantly showed activation by mechanical pain in our investigation.

Summarizing, we could show pronounced activations in the dorsal parts of the thalamus (mainly medio-dorsal and to a lesser extent ventro-latero-dorsal) by mechanical pain, proposing that mechanical pain is in fact processed by the thalamus.

### Heat pain

Heat pain did not result in thalamic activations in our patient group, even though the stimulation was painful in all participants. The utilized heat pain temperatures were well above the pain thresholds based on standard data for men and women younger than 40 described by Rolke et al. (2006). The analysis of the first 3 s of heat stimulation, examined to overcome habituation processes (Treede et al. 1995), also did not result in BOLD responses within the thalamus. The same protocol to elicit heat pain has been used in prior experiments of our study group and evoked reliable activations (e.g., of the insula, ACC, and S1) but not within the thalamus (Habig et al. 2017; Schirner et al. 2014). In a multivariate pattern analysis to decode the perception of laser-evoked pain, no significant thalamic activation occurred during laser-evoked pain stimulation or anticipation of pain (Brodersen et al. 2012). Moreover, Peltz et al. (2011) did not observe thalamic activation by noxious heat stimulation performing fMRI. Therefore, we postulate that heat pain is barely processed via the thalamus.

Iannetti et al. (2005) combined laser-evoked potentials (by an Nd:YAP laser) to distinctly stimulate Aδ fibres with fMRI. The published images show no thalamic activation.

Concordant with this thesis, Peyron et al. (2000) already discussed the way bithalamic activation, which has been observed throughout pain experiments with heat pain, relies on “attentional and vigilance processes” rather than on sensory processing. Geuter et al. (2013) only found thalamic activation in a late interval (10–20 s), underpinning this assumption. Furthermore, Sprenger et al. (2015) showed only posterior thalamic activations when comparing high-intensity heat pain (47 °C, NRS mean = 56.7) with low-intensity heat pain (46 °C, NRS mean = 29.8), indicating a role in heat pain evaluation rather than heat pain perception.

### Multimodal signalling of thalamic nuclei

All stimuli applied to the left body side (except heat pain) showed overlapping BOLD responses in the anterior to medial parts of the thalamus, namely the right anterior and ventral-anterior nucleus and the left medio-dorsal nucleus. This finding might imply that these nuclei are multisensory nuclei for processing multimodal sensory information such as touch, pain, and vestibular stimulation.

Cauda et al. (2012) found the medio-dorsal thalamus to be involved in pain and touch as well as attentional and reward tasks. The same applied to the anterior insula and the dorsal anterior cingulate cortex; the authors, therefore, postulated a thalamic contribution to attentional and salience processes. Congruently, the anterior part of the thalamus has been attributed to the limbic system by fibre-tracking techniques (Grodd et al. 2020; Kumar et al. 2015).

Beyond that, our data indicate that thalamic nuclei have multimodal functions as previously postulated by other study groups (Behrens et al. 2003; Hwang et al. 2017; Johansen-Berg et al. 2005; Wijesinghe et al. 2015) because all applied distinct stimuli showed overlapping BOLD responses within the thalamus. It needs to be taken into account that we cannot provide evidence for intranuclear connections within the thalamus by observation of BOLD responses (also see “Limitations”). But nevertheless, our finding is in line with Kumar et al. (2015), who showed that functional connections of thalamic parcels (defined by DTI) could not be dedicated to certain cortical areas or lobes underpinning the thesis of multimodality of thalamic nuclei.

Accordingly, a recent functional connectivity study showed extensive connections to and from the thalamic nuclei, confirming that one thalamic nucleus receives input from diverse cortical areas with multiple functions and likewise projects to many other cortical areas. The thalamus has, therefore, been described as an “integrative hub for functional brain networks” (Hwang et al. 2017). This theory has been confirmed by human lesion studies (Hwang et al. 2021).

Jones argued that there is no anatomical evidence for connections between dorsal thalamic nuclei (Jones 2007). He describes a basic bidirectional circuitry of connections between “afferent fibers, thalamocortical relay cells, intrinsic interneurons, reticular nucleus cells and the cerebral cortex” and shows two classes of corticothalamic neurons: (1) corticothalamic neurons with somata in layer VI that mainly project to distinct thalamic nuclei; (2) corticothalamic neurons with somata in layer V of the cerebral cortex that project to different but functionally related thalamic nuclei.

In addition, not only could overlap of BOLD responses by distinct sensory stimuli be observed but also a time-dependent activation of clusters within the thalamus. Early stimulation mainly exceeded the late stimulation interval, matching the “gatekeeping” function of the thalamus (Newman 1995). However, we could observe the activation of different thalamic nuclei when assessing the whole time interval or the spilt time intervals, providing evidence for a network of signal processing within the thalamus.

### Lateralization

It remains unclear why stimuli applied to the left body side dominated right-sided stimulation. The same stimulus intensities were applied bilaterally, and we performed the experiment in randomized order, beginning on the left or the right hand to overcome habituation. The investigator did not change throughout the experiment.

Hypothetically, the dominant side reacts to a lesser extent to sensory stimuli because input to the dominant hand is more experienced in central processing, but there is no profound evidence for this thesis in the literature. Therefore, we need to consider constructional bias. There was much more space around the side of the scanner where the participants placed their left arm, potentially causing a slightly different angle from the investigator to the participant. Nevertheless, galvanic stimulation and heat pain were triggered outside the scanner room.

### Habituation

All sensory stimuli (except heat pain) showed stronger activations with larger clusters at the early time interval (T1, 0–3 s). This fits well with the theory that the thalamus is a gatekeeper of sensory stimuli: With longer-lasting stimuli, the thalamus shows less activity, possibly because the stimulus is already being processed in higher brain areas. Another plausible reason might be pre-thalamic habituation or stimulus depression on the spinal level.

A preponderance of activations during the first seconds of the stimuli might correlate to the first perceiving of stimuli and shift to evaluating these sensations. Equivalent observations have been reported by zu Eulenburg et al. (2013).

### Limitations

An analysis of differences in stimulus modalities can be biased by different intensities of all applied stimuli. This is not applicable to the observed side differences because we performed all stimuli with equal intensities on both hands and vestibular organs.

We cannot explain the dominance of stimuli applied to the left body side in comparison to right-sided stimulation. However, because we aimed to present a topographic map of activation by distinct stimuli of the thalamus rather than laterality of stimulation, this observation does not disturb the aim of the study.

Due to spatial smoothing with 5 mm FWHM, activation in smaller regions might have been overlooked.

Since functional MRI and not fiber tracking techniques were employed, we can only describe overlapping BOLD signals but cannot proof thalamic intranuclear connections.

## Conclusions

We present new evidence for thalamic participation in the encoding and integration of different sensory modalities. We postulate that thalamic nuclei possess multimodal functions because the registered BOLD responses showed overlapping clusters for the different sensory modalities (galvanic stimulation, light touch, and mechanical pain). Besides that, we observed an accessory topographic order within the thalamus with activation by galvanic stimulation lateral and ventral to mechanical pain. The time-dependant activation difference within the thalamus underpins the role of the thalamus as a gatekeeper for sensory information. Connectivity analysis might further explore the complicated thalamo-cortical networks.

## Data Availability

Due to the local ethics committee statement in this study, survey respondents were assured raw data would remain confidential and would not be shared for public use. On individual requests, we can provide including raw and processed data sets.
